# Seasonal variation in daily patterns of social contacts in the European badger *Meles meles*

**DOI:** 10.1002/ece3.3402

**Published:** 2017-09-25

**Authors:** Matthew J. Silk, Nicola Weber, Lucy C. Steward, Richard J. Delahay, Darren P. Croft, David J. Hodgson, Mike Boots, Robbie A. McDonald

**Affiliations:** ^1^ Environment and Sustainability Institute University of Exeter Penryn UK; ^2^ Centre for Ecology and Conservation University of Exeter Penryn UK; ^3^ National Wildlife Management Centre Animal and Plant Health Agency Gloucestershire UK; ^4^ Centre for Research in Animal Behaviour University of Exeter Exeter UK; ^5^ Integrative Biology University of California Berkeley CA USA

**Keywords:** bovine tuberculosis, diel cycle, proximity logger, seasonal forcing, social dynamics

## Abstract

Social interactions among hosts influence the persistence and spread of infectious pathogens. Daily and seasonal variation in the frequency and type of social interactions will play an important role in disease epidemiology and, alongside other factors, may have an influence on wider disease dynamics by causing seasonal forcing of infection, especially if the seasonal variation experienced by a population is considerable. We explored temporal variation in within‐group contacts in a high‐density population of European badgers *Meles meles* naturally infected with *Mycobacterium bovis* (the causative agent of bovine tuberculosis). Summer contacts were more likely and of longer duration during the daytime, while the frequency and duration of winter contacts did not differ between day and night. In spring and autumn, within‐group contacts peaked at dawn and dusk, corresponding with when they were of shortest duration with reduced potential for aerosol transmission of pathogens. Summer and winter could be critical for transmission of *M. bovis* in badgers, due to the high frequency and duration of contacts during resting periods, and we discuss the links between this result and empirical disease data. This study reveals clear seasonality in daily patterns of contact frequency and duration in species living in stable social groups, suggesting that changes in social contacts could drive seasonal forcing of infection in wildlife populations even when the number of individuals interacting remains similar.

## 1 INTRODUCTION

The social behavior of animals can vary across space and time in a predictable manner (Sueur et al., 2011; Silk, Croft, Tregenza, & Bearhop, 2014). In particular, for many species, there may be considerable seasonal variation in the drivers of social and spatial behavior that result in substantial differences in how they interact with conspecifics (Couzin, 2006; Sueur et al., 2011; Silk et al., 2014). This seasonal variation in the nature of social interactions could have important implications for dynamic processes occurring within these populations, such as disease transmission (Altizer et al., 2006; White, Forester, & Craft, 2017). The latter is of particular interest as seasonal forcing of infection can play an important role in infectious disease dynamics (Altizer et al., 2006; Grassly & Fraser, 2006). Seasonality in disease transmission caused by variation in social behavior has been documented in several wildlife populations (Hosseini, Dhondt, & Dobson, 2004; Altizer et al., 2006; Begon et al., 2009; Duke‐Sylvester, Bolzoni, & Real, 2011) and is known to have important implications for disease dynamics. However, studies examining this phenomenon have typically focused on the role of seasonal reproduction (Hosseini et al., 2004; Duke‐Sylvester et al., 2011) or substantial changes in sociality over the course of the annual cycle (Hosseini et al., 2004). In contrast, there has been little research on how seasonality (both through its direct effect on behavior or indirectly through its effects on the ecological environment) may influence fine‐scale patterns of social interaction, for example within relatively stable social groups.

Although temporal dynamics in social contacts can be integral to the epidemiology of infectious diseases (Craft, 2015; White et al., 2017), it is only recently that technological developments have facilitated their quantification in wild animals (Drewe et al., 2012; Krause et al., 2013). For the spread of infection, some types of interaction are likely to be more important than others (Blyton, Banks, Peakall, Lindenmayer, & Gordon, 2014; Craft, 2015; White et al., 2017), and hence, variation in the nature of social contacts may be an important driver of seasonal transmission dynamics (Hamede, Bashford, McCallum, & Jones, 2009; Reynolds, Hirsch, Gehrt, & Craft, 2015; Hirsch, Reynolds, Gehrt, & Craft, 2016). For infections that are spread via aerosol transmission, simultaneous peaks in the frequency and duration of interactions are likely to correspond with heightened transmission risks. This will be especially apparent if transmission is disproportionately more likely from longer interactions. In this case, periods with relatively frequent interactions of long duration may provide many more transmission opportunities than periods with frequent but short duration interactions. While bio‐logging data does not necessarily identify the nature of social interactions, it does indicate how the duration and frequency of interactions varies over time, and this is likely to serve as an important proxy for transmission risk.

**Figure 1:**
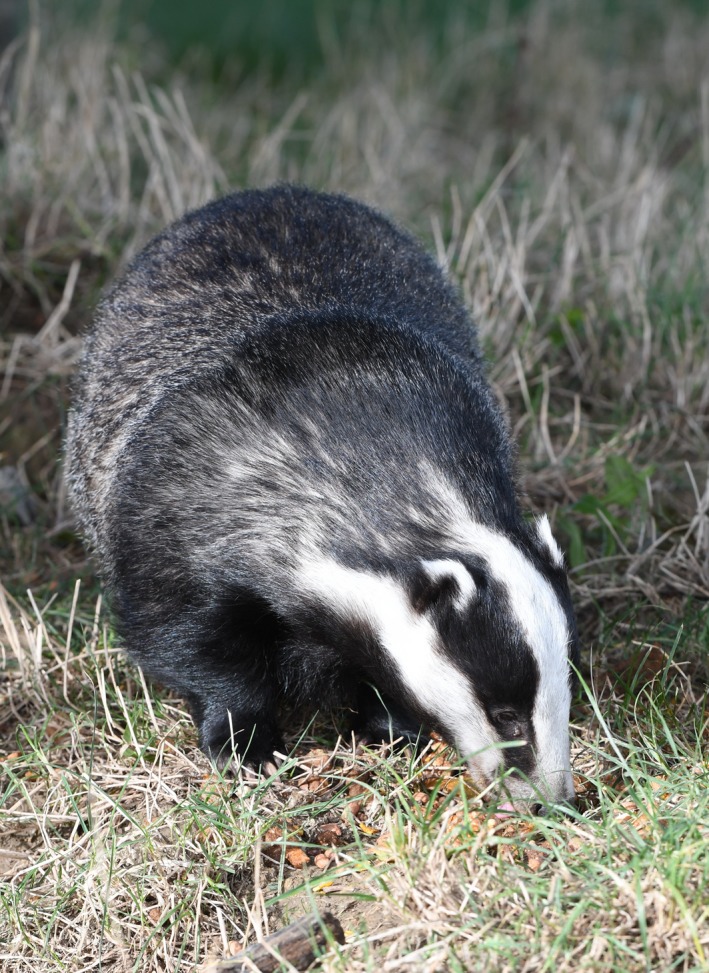
A European badger *Meles meles*

We used proximity loggers to explore daily and seasonal patterns of social contacts for 1 year in a high‐density population of European badgers *Meles meles* (Figure 1) naturally infected by *Mycobacterium bovis*, the causative agent of bovine tuberculosis (bTB). While proximity loggers cannot provide information on the exact nature of social interactions that take place, features of a proximity event (“social contact”) such as its duration can be used as a proxy for transmission opportunities. Badgers are an important wildlife reservoir of *M. bovis* and a source of infection for cattle in the United Kingdom and Ireland (Donnelly et al., 2006; Godfray et al., 2013). This global disease of livestock is a persistent economic problem in these countries (Godfray et al., 2013). Badgers are nocturnal, foraging asocially at night and resting in communal burrow systems (setts) during the day (Roper, Ostler, Schmid, & Christian, 2001). In high‐density populations, badgers live in social groups inhabiting shared setts (Roper, 2010), frequently interacting with individuals from their own social group, but with fewer interactions with individuals from other groups (Weber et al., 2013; O'Mahony, 2015). Transmission of bTB among badgers is thought to occur chiefly via aerosol (Cheeseman, Wilesmith, & Stuart, 1989; Weber et al., 2013), although there is also evidence that biting may also be implicated (Jenkins, Cox, & Delahay, 2012) and that infection can be acquired from the environment (Courtenay et al., 2006; King et al., 2015).

There is considerable seasonal variation in territoriality, reproductive behavior and activity levels across the annual cycle of the European badger (Roper, 2010), which generates seasonal variation in social contact network structure (Weber et al., 2013; Silk et al., 2017), and we expected that this would be reflected in seasonal variation in daily contact patterns. We also anticipated that seasonal variation in contact patterns might be correlated with seasonal differences in the likelihood of individuals becoming infected (Gallagher & Clifton‐Hadley, 2000; Buzdugan, Vergne, Grosbois, Delahay, & Drewe, 2017). It is likely that there is additional seasonal variation in individual state among these same periods (e.g., Audy et al., 1985; George, Smith, Mac Cana, Coleman, & Montgomery, 2014; Rogers, Cheeseman, & Langton, 1997), and we acknowledge that other factors such as this may play additional roles. More specifically, we predicted that social contacts would be less frequent in the spring when females have dependent cubs (Roper, 2010) and males are engaged in territorial behavior (Roper et al., 1993) than in other seasons, particularly as a similar pattern had been previously demonstrated in a medium‐density population of badgers (O'Mahony, 2015). We also expected to observe increased contact duration during the day when badgers are resting in communal setts, and dawn and dusk peaks in contact frequencies (O'Mahony, 2015) reflecting emergence from and return to the sett. However, we predicted considerable seasonal variation in these trends. While badgers do not hibernate, they become much less active during winter, especially when temperatures are low (Lindsay & Macdonald, 1985; Woodroffe & Macdonald, 1995; Roper, 2010). Therefore, we predicted less daily variation in contact frequency and duration in winter than in summer. Seasons are likely to be especially important for aerosol transmission of *M. bovis* if there are concurrent peaks in contact frequency and duration (i.e., many instances of prolonged close contact). In studies of the same badger population, diagnostic test results suggested a peak in the acquisition of infection during winter (Gallagher & Clifton‐Hadley, 2000; Buzdugan et al., 2017), and concurrent peaks in contact frequency and duration occurred during this period would be consistent with a potential role for seasonal changes in social behavior in contributing to this pattern.

## 2 MATERIALS AND METHODS

### 2.1 Study system and data collection

We deployed proximity‐logging radio tags (Sirtrack, Havelock North, NZ) on 51 free‐living badgers (24 males, 27 females) at Woodchester Park, Gloucestershire, UK (51°71′N 2°30′W), between June 2009 and May 2010 (see Weber et al., 2013). Woodchester Park is 7 km^2^ of deciduous and coniferous woodland on the Cotswold escarpment surrounded by mixed agricultural land. The area has a temperate climate with four distinct seasons (summer, autumn, winter, and spring). Day length and temperatures are highest during the summer (mean temperature from 1989 to 2014: 16.04 ± 0.13°C), and day lengths shortest and temperatures lowest during the winter (mean temperature from 1989 to 2014: 4.68 ± 0.23°C). The proximity devices transmit unique ultra‐high frequency (UHF) codes and detect and record the identity of one another. Proximity (“social contact”) is detected when loggers were within 0.64 ± 0.04 m of one another, a distance within which *M. bovis* transmission is likely to be possible (Weber et al., 2013). Tagged individuals were from nine main setts in the core study area of the population and represented approximately 80% of the nonjuvenile individuals from these setts (Weber et al., 2013). We separated individuals into six social groups for the purpose of this study on the basis of the results of a multilevel community detection algorithm run on the full annual population social network in the R package igraph (Csardi & Nepusz, 2006). Using this approach directly relates group membership to social contacts and incorporates any changes in spatial behavior over the course of the study. In total 59 collars were used in the study (on 51 individuals) as some collars were replaced if they were lost or became damaged. Individuals varied with respect to how long they were collared for, but we controlled for any affect that this might have had on the relative performance of the collars (Drewe et al., 2012) by including duration of collaring in any analyses where we compared between different months or seasons.

### 2.2 Data analysis

**Figure 2:**
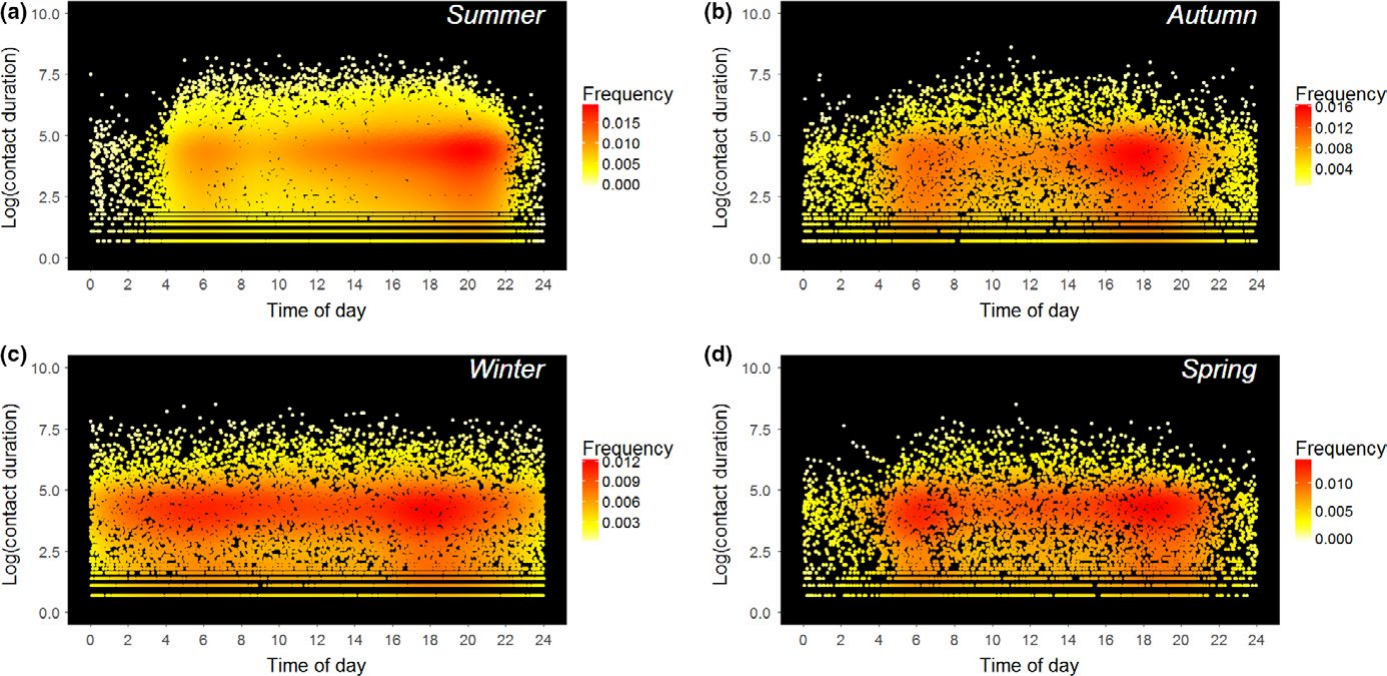
Seasonal differences in the daily pattern of contact frequency and duration in a high‐density population of European badgers. The value of each contact on the y axis is the natural logarithm of its duration. The plots show raw contact data for (a) summer (June–August), (b) autumn (September–November), (c) winter (December–February), and (d) spring (March–May). The shading represents the frequency of contacts within a local region of the graph, with red representing the highest frequency of contacts in a particular region (i.e., combination of time of day and duration) and pale yellow the lowest

Contact data were processed using established methods by joining contacts within a 90‐sec threshold and removing additional 1‐sec contacts (Drewe et al., 2012). Any day on which an individual was physically captured, and the following 2 days, was excluded from the analyses. Data were processed to remove duplicate contact events by only including data from the alphabetically first individual if collars were retrieved from both individuals. To ensure that this did not affect our results, we also conducted the same analyses when only the alphabetically second individual was used and the results did not differ qualitatively. Analysis of annual patterns of contact frequency (both seasonal and monthly) was conducted between collars rather than individuals to enable the time since a collar was deployed to be accounted for in the model. The distributions of the frequency and duration of all contacts in different seasons after data processing are displayed in Figure 2. In total 60,108 contacts were included in the analysis, of which 58,228 (96.9%) were within social groups. As a result, while we included extra‐group contacts within analysis of daily patterns of contact frequency and duration, we focused on within‐group contacts when analyzing annual variation in contact frequency. It is clear that within‐group contacts typically make up the vast proportion of an individual's social interactions in this population, and the importance of irregular extra‐group contacts has been well described by previous social network analyses (Weber et al., 2013).

### 2.3 Seasonal analysis of contact frequency

Seasonal patterns of contact frequency were calculated at a dyadic level (i.e., separately for each pair of collars) meaning that some individuals were represented by multiple collars within a single season. Seasons were defined as summer: June–August, autumn: September–November, winter: December–February, and spring: March–May. The number of contacts recorded in each season was 27,742, 10,302, 14,252, and 7,812, respectively. Raw contact data were used to generate a mean contact frequency for each dyad of collars within a season. This was the total number of contacts divided by the number of days that both collar IDs were contemporaneously functioning (excluding day of capture and 2 days after). The probability of a contact occurring for each within‐group dyad (one if a contact did occur and zero otherwise; binomial error distribution) and the frequency of contacts within a dyad if contact did occur (number of contacts divided by number of days; log transformation and Gaussian error distribution) were modeled with season (summer, autumn, winter, and spring), social group, sampling effort, and the length of time each individual had been collared as fixed effects and the identity of each collar in a dyad as two random effects (Table 1). Models were run in R 3.3.0 (R Development Core Team 2017) using the package lme4 (Bates, Maechler, & Bolker, 2012). Model estimates and statistical significance of fixed effects were inferred from the full model. The sampling effort term was the number of days that both collar IDs in a given collar dyad were functioning contemporaneously. Two lengths of time collared terms were included in each model (probability of contact and contact frequency), one for each collar within a dyad. These terms reflected the number of days a collar had been deployed at the start of a given season. For example, a collar fitted on 29 May 2009 would have a length of time collared term of 2 for summer, 94 for autumn, 185 for winter, and 275 for spring. Collars fitted half way through a season could have a negative value for length of time collared. For example, a collar fitted on 27 October 2009 would have a length of time collared term of −56 for autumn, 35 for winter, and 125 for spring. As we have highlighted elsewhere (Drewe et al., 2012), a negative correlation between length of time collared and contact frequency might be expected due to a deterioration in battery performance that results in a reduced probability of longer range contacts being detected. Histograms of daily patterns of contact frequency were constructed separately for each season by calculating frequencies in 30‐min time intervals.

**Table 1:** Models to test which factors influence seasonal variation in badger contacts

Model	Model terms	Type of effect	Reason for inclusion
Contact probability	Season	Fixed	To test for differences in contact probabilities in different seasons
	Social Group	Fixed	To test for differences in contact probabilities between the six social groups
	Sampling Effort (days both individuals collared)	Fixed	To control for the length of time both individuals in a dyad were collared (each month)
	Length of time collared (days since collar deployment)	Fixed	To control for deterioration in collar performance over time [13]
	Individual ID 1	Random	To account for individual variation
	Individual ID 2	Random	To account for individual variation
Contact frequency	Season	Fixed	To test for differences in contact frequency in different seasons
	Social Group	Fixed	To test for differences in contact frequency between the six social groups
	Sampling Effort	Fixed	To control for the amount of time two individuals could have interacted within a given month
	Length of time collared	Fixed	To control for deterioration in collar performance over time. One term for each collar in a dyad.
	Individual ID 1	Random	To account for individual variation
	Individual ID 2	Random	To account for individual variation
Contact duration	Season	Fixed	To test for differences in contact duration between seasons
	Time of day^4^	Fixed	To test for changes in contact duration over a day. The fourth‐order polynomial allowed three points of inflexion to incorporate crepuscular behavior.
	Season × Time of day^4^	Fixed	To test for differences in the daily pattern of contact duration among seasons
	Social Group	Random	To account for variation among social groups
	Individual ID 1	Random	To account for individual variation
	Individual ID 2	Random	To account for individual variation

The fixed and random effect structure of the three generalized linear mixed effects models are provided, together with the reasons for inclusion of each term.

### 2.4 Monthly analysis of contact frequency

To investigate the robustness of the seasonal differences in contact patterns observed and to explore more fine‐scale variation within these seasonal patterns, the contact frequency models were rerun with month as an explanatory variable instead of season. The analysis used an otherwise identical set of fixed and random effects (Table S1). Histograms of daily contact patterns were then also constructed by month rather than by season, using the same 30‐min time intervals as per the seasonal analysis.

### 2.5 Seasonal analysis of contact duration

The relationship between contact duration and the interaction between season and a fourth‐order polynomial effect of time of day was modeled using a linear mixed effects model (log‐ transformed response variable, Gaussian residuals) using the R package lme4 (Bates et al., 2012). Social group and the identity of each individual in a dyad were included as random effects in the model (see Table 1). Time of day was standardized across the year so that 25% of the day elapsed before sunrise, 50% between sunrise and sunset, and 25% after sunset. The fourth‐order polynomial for time of day optimized AIC (Aikaike information criterion) values and enabled crepuscular changes in activity to be modeled. To confirm that using a fourth‐order polynomial was appropriate, we also fitted two general additive models (GAMs) to the same dataset using the R package mgcv (Wood, 2001), one using the same standardized time of day and one using the time of day in seconds. In these models, social group and the identity of each individual in a dyad were included as fixed effects. The length of time collared was not included as a fixed effect in any of these models as we were interested in differences in daily patterns rather than between the seasons themselves, and therefore, deterioration in collar performance would not be expected to have the same influence on the results.

## 3 RESULTS

The raw patterns of contact frequency and duration are displayed together in Figure 2. Neither season (χ^2^_(3)_ = 2.91, *p* = .41) nor social group (χ^2^_(5)_ = 3.47, *p* = .63) influenced the probability of occurrence of within‐group contacts. The frequency of recorded contacts did, however, vary with season (χ^2^_(11)_ = 15.03, *p* = .002) but not among social groups (χ^2^_(5)_ = 7.18, *p* = .21). Contact frequency peaked in summer (June–August), was similar in autumn (September–November) and winter (December–January) and was lowest in spring (Table 2). Analysis by month showed a secondary, smaller peak in contact frequency in December and January (Tables S1 and S2). Sampling effort did not affect the probability of contact (0.001 ± 0.005; χ^2^_(1)_ = 0.06, *p* = .81) or contact frequency (−0.002 ± 0.003; χ^2^_(1)_ = 0.37, *p* = .54). The length of time individuals had been collared (controlling for decline in logger performance) did not affect contact probability (ID1: χ^2^_(1)_ = 3.01, *p* = .08, ID2: χ^2^_(1,22)_ = 0.29, *p* = .59) but did reduce contact frequency (ID1: χ^2^_(1)_ = 5.73, *p* = .02, ID2: χ^2^_(1)_ = 0.07, *p* = .80). As expected, collars that had been deployed for longer detected fewer contacts (model estimate: −0.003 ± 0.001), although this effect was limited to the primary individual in each dyad.

**Table 2:** The effect of season on the probability of within‐group contacts in badgers and their mean daily frequency if they do occur

Season	Mean probability of a contact event	Mean daily frequency of contacts
Summer	0.75 (0.50–0.91)	2.93 (1.47–5.85)
Autumn	0.72 (0.45–0.89)	1.18 (0.57–2.41)
Winter	0.76 (0.48–0.92)	1.26 (0.58–2.75)
Spring	0.84 (0.56–0.95)	0.61 (0.25–1.48)

Model predictions are back‐transformed model estimates with standard errors, for dyads in group one simultaneously collared for 90 days of a season and for a time since collared of zero days.

**Figure 3:**
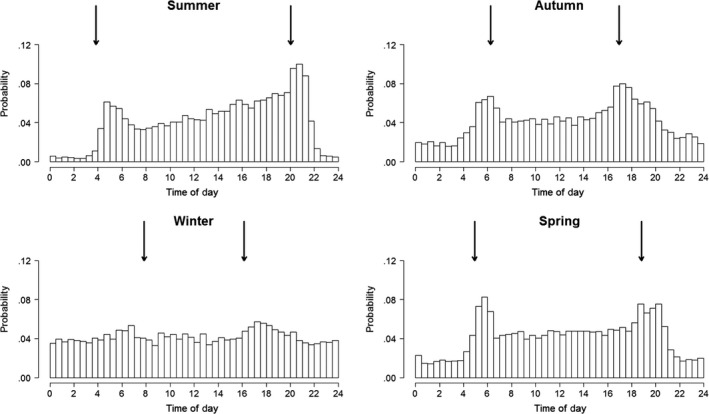
Seasonal variation in within‐group contact frequency of badgers (summer: June–August, autumn: September–November, winter: December–February, and spring: March–May). Arrows indicate sunrise and sunset times on the middle day of each season. Each day is split into 30‐min intervals

Daily patterns of within‐group contacts varied throughout the year (Figures 2 and 3, and Fig. S1). From spring until autumn, contacts were far more frequent during daylight hours, especially in summer. There was a small peak in contact frequency shortly after dawn and a higher peak shortly after sunset. During spring and autumn, these dawn and dusk peaks in contact frequency were similar in magnitude, and the difference in contact frequency between day and night was generally smaller. Diel patterns were weak during winter, especially December and January (Fig. S1). During the summer, there was a tendency for contact frequency to increase throughout the course of the daytime resting period, so that more contacts were recorded later in the afternoon than during the morning. This pattern was not apparent during other seasons.

**Figure 4:**
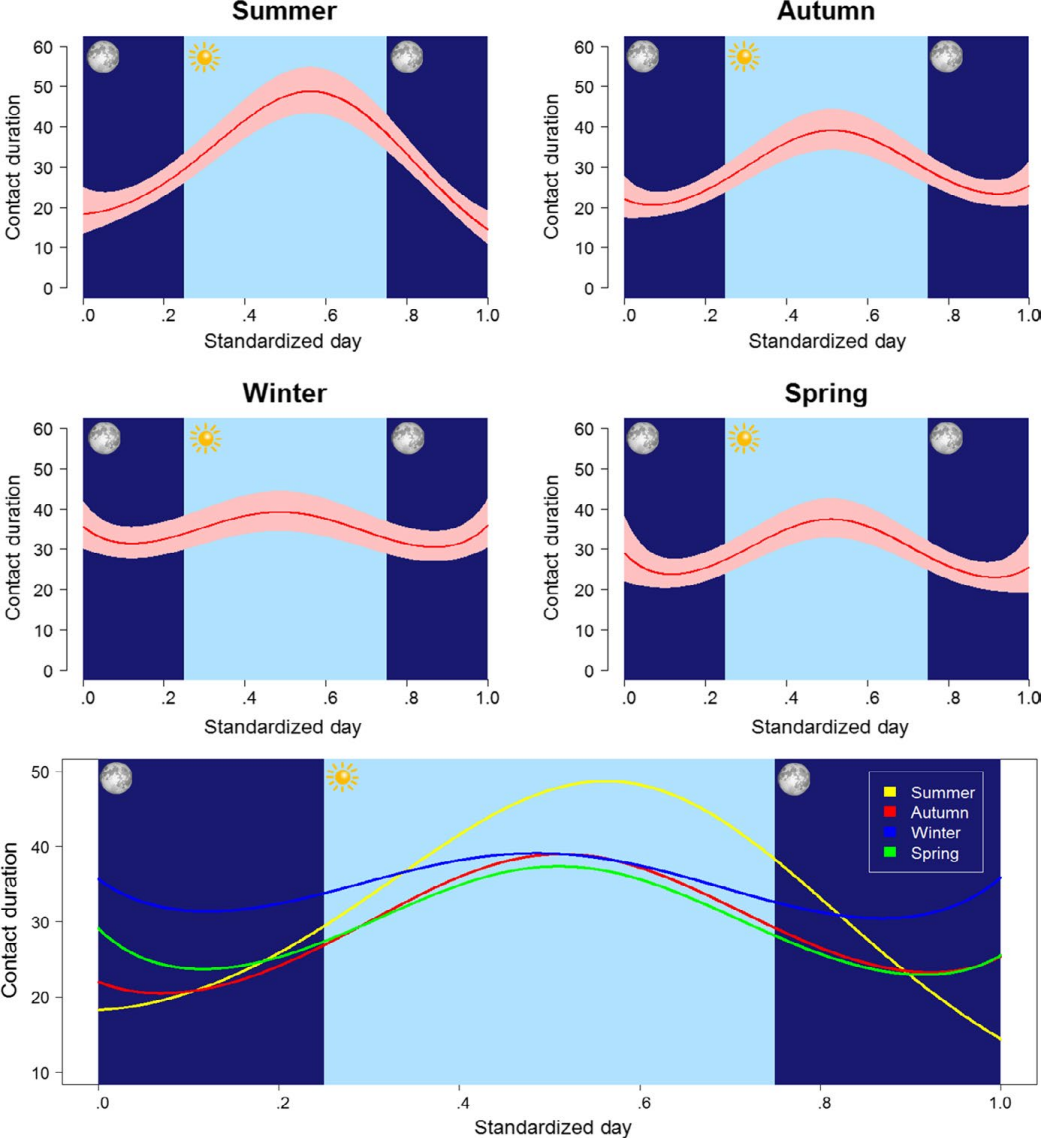
Seasonal differences (summer: June–August, autumn: September–November, winter: December–February, and spring: March–May) in daily patterns of contact duration among badgers. In the top four plots, the red line represents model predictions and the red shaded area their 95% confidence intervals. The bottom panel shows model predictions for each season together. Day has been standardized so that 50% (between 0.25 and 0.75) of a 24‐hr period is between sunrise and sunset at all times of year

Daily patterns in contact duration differed among seasons (test of interaction: χ^2^_(12)_ = 154.05, *p* < .001; Figure 4). During summer, within‐group contacts were substantially longer during the day than at night (Figure 4a). However, during winter, contacts were of similar duration throughout day and night (Figure 4c). In spring (Figure 4d) and autumn (Figure 4b), there was a small peak in contact duration during the day. These results were supported by the output of both GAMs (Figs. S2 and S3). When standardized time of day was used, there was an apparent increase in contact duration in the early hours of the morning during summer (Fig. S2), but this was not present when nonstandardized time of day was used as a response variable so was likely an artifact of the model fitting.

## 4 DISCUSSION

We reveal substantial daily and seasonal variation in contact patterns in a high‐density population of badgers, with potentially important implications for seasonal forcing of *Mycobacterium bovis* transmission risk. Furthermore, these patterns can be related to the relatively well‐understood annual cycle of European badgers and highlight the importance of seasonal behavior in generating variation in the frequency and nature of contacts in social animals.

The daily patterns of social contacts observed in this study were broadly similar to those found in a medium‐density badger population by O'Mahony (2015), and it was evident that contact frequency was distinctly seasonal, with extremes of variation in summer and winter. In summer, contacts were much more frequent during daylight hours when badgers are resting in communal setts and very rare at night when badgers are active (Roper, 2010). In contrast, the frequency of social contacts in winter remained similar throughout the 24‐hr period. Both spring and autumn are somewhat intermediate in this regard. In spring, summer, and autumn (March–November), there were peaks in contact frequency at dusk and dawn that are likely to be related to the emergence of individuals from setts at the start of a night, and then their subsequent return to the sett. During these periods, the detection of social contacts may simply reflect overlap in the activity of individuals and be more likely to reflect proximity rather than true social interactions, as emergence from and return to the sett represent a likely bottle‐neck at which most individuals could potentially come into close proximity. A final interesting pattern revealed by proximity data was that during the summer months (principally May–August), there was a tendency for contact frequency to increase through the daylight period so that contact frequency was substantially higher in the late afternoon than morning. While we collected data for one 12 month period, weather patterns were largely as expected. Therefore, these results suggest considerable seasonal variation in daily patterns of contacts that are robust to more fine‐scale weather‐related variation in badger activity (e.g., Noonan et al., 2014).

In summer, the low frequency of night‐time contacts is likely caused by reduced reproductive and territorial behavior, and individuals ranging further to forage than during other seasons (Roper, 2010). When ranging over wider areas, social contact with individuals from the same social group would be expected to become less likely. In addition, during summer, outlying setts are used more frequently by some individuals (Weber et al., 2013), and in main setts, some badgers only tend to share chambers with particular associates (Roper et al., 2001). Together, this may result in asymmetries in the increase in contact frequency, so that contact frequencies are substantially higher within certain dyads but not more generally. The reduced tendency for badgers to share sett chambers during summer (Roper et al., 2001) may also explain the increase in contact frequency in the late afternoon and evening if individual badgers start moving around the sett before they emerge (e.g., Noonan et al., 2015).

Given the well‐documented reduction in badger activity during winter (Lindsay & Macdonald, 1985; Woodroffe & Macdonald, 1995; Noonan, Rahman, Newman, Buesching, & Macdonald, 2015), daily patterns of contact duration in the current study changed as expected from summer through to winter. During the summer months, there was also a substantial peak in contact duration during the daytime, while in winter contact duration remained similar throughout the daily cycle. Spring and autumn were intermediate with smaller peaks in contact duration than summer. High duration contacts are likely to represent social interactions taking place within or in close proximity to setts. The proximity‐logger data collected during the present study suggests that previously established reductions in activity during winter have a substantial influence on daily patterns of social dynamics. During winter, badgers are much less likely to use outlying setts (Weber et al., 2013) and more likely to share chambers within a main sett (Roper et al., 2001), meaning that increases in contact frequency and duration are likely to be spread more evenly between dyads than during summer months. The 2009/2010 winter at Woodchester Park was considerably colder than average (mean December–February temperature 2.40°C), which would be expected to result in reduced activity (Lindsay & Macdonald, 1985), and may result in the differences in daily patterns in contact from other seasons being greater than normal. However, even in a warmer winter, reduced activity is likely to result in a qualitatively identical trend.

Our study also revealed overall seasonal differences in contact frequencies, which were at their lowest in spring, highest in summer, and intermediate in autumn and winter. This finding is largely supportive of the previous work showing that within‐group network strength (the total sum of contact durations with groupmates) was highest in summer and lowest in autumn and spring (Weber et al., 2013). In spring, contact rates might be reduced as a result of reproduction, with females with dependent cubs highly unlikely to share chambers with other adult badgers. The autumn, winter, and spring results match closely with those of O'Mahony (2015), despite differences in approach such as our attempt to control for deterioration in collar performance over the course of their deployment (Drewe et al., 2012). This suggests that these patterns may be both robust to any battery‐related effects on the contacts being recorded and generally observed regardless of badger population density. However, in contrast to the study by O'Mahony (2015), we were also able to record contact frequencies during the summer months, and these were substantially higher than during any other season, even while controlling for deterioration in collar performance.

### 4.1 Links to empirical disease data

Two previous studies have investigated seasonal trends in bTB infection in badgers at a population level in the population used in the present study. Gallagher and Clifton‐Hadley (2000) identified a winter peak in the number of incident cases, and a secondary summer peak that they described as a likely artifact. A more recent study using Bayesian modeling of diagnostic test results reported that individual badgers were more likely to transition from negative to positive bTB status in the winter and spring (Buzdugan et al., 2017). While we are unable to directly relate our recorded changes in social contacts to infection, both of these studies point to winter as a likely key time for bTB transmission among badgers. There are likely to be two possible explanations for this; either badger behavior at this time increases exposure to *M. bovis* or some aspect of their physiology (e.g., body condition, immuno‐competence) makes individuals more susceptible to the pathogen. Despite a wealth of information on badger ecology, there is limited information about the latter of these two possibilities. Badgers tend to be in better body condition during winter than in summer (Rogers et al., 1997). Changes in hormones that might alter immuno‐competence and susceptibility also provide mixed evidence, as although testosterone in males peaks in late winter (Audy et al., 1985), levels of cortisol show spring or summer peaks and are negatively correlated with body condition (George et al., 2014). There is currently more compelling evidence to support an increase in *M. bovis* exposure risk driven by seasonal changes in badger behavior, given that the empirical data available point toward neither stress physiology or body condition providing a likely explanation. However, this is subject to further studies that explore other components of seasonal variation in physiological state, especially immune‐competence. For example, the present study shows a slight secondary peak in contact frequency during the winter months, combined with consistent contact frequency and duration during day and night. While, it is not possible to identify the exact nature of social interactions using proximity‐logger data, longer duration contacts most probably relate to underground interactions in setts, especially during periods of inactivity. Therefore, while not all of these long duration, underground social contacts may provide transmission opportunities, their protracted nature in a confined space would be expected to provide a better opportunity in general for *M. bovis* transmission (Cheeseman et al., 1989; Weber et al., 2013). Also, the tendency for individuals to use main setts more and occupy a smaller area within the sett during winter (Roper et al., 2001; Weber et al., 2013) may contribute to this enhanced exposure risk if it resulted in an increased density and/or reduced path length within social group contact networks (see Silk et al., 2017), or alternatively meant that individuals spent more time in contaminated environments. Another possible source of enhanced risk is bite‐wounding, which is a suspected transmission route in some populations (Jenkins et al. 2012), is also typically most frequent during winter months (Delahay et al., 2006). Finally, as contamination of the environment (with badger feces and urine) may be a potential source of exposure to *M. bovis* (Courtenay et al., 2006; King et al., 2015), increased use of main setts and reduced ranging in winter could have the effect of concentrating such transmission risks.

### 4.2 Seasonal behavior and disease epidemiology in wildlife

The coincidence of observed seasonal changes in daily patterns of social contacts and increased bTB incidence in this high‐density badger population is notable as it is consistent with seasonal forcing of infection (Altizer et al., 2006; Grassly & Fraser, 2006). Although relatively few studies have investigated the role of seasonal changes in host behavior in driving long‐term epidemiological patterns in wildlife populations, some have identified significant effects (see Hosseini et al., 2004; Altizer et al., 2006; Begon et al., 2009; Duke‐Sylvester et al., 2011). However, in these systems, seasonal changes in behavior have been found to have an important influence on long‐term disease dynamics. For example, Hosseini et al. (2004) showed that in house finches *Haemorhous mexicanus,* observed dynamics *of Mycoplasma gallisepticum* infection were best explained by seasonal forcing as a result of both flocking during the winter and seasonal breeding. While in raccoons *Procyon lotor*, increased seasonal forcing of rabies infection resulted in spatially asynchronous epidemics (Duke‐Sylvester et al., 2011). The impact of seasonal forcing can be particularly apparent for pathogens with low R_0_, as it may generate periodicity in prevalence that would otherwise not occur (Bolzoni, Dobson, Gatto, & De Leo, 2008). Given that *M. bovis* has a low R_0_ in badgers (Delahay et al., 2013) and patterns of bTB prevalence are spatially asynchronous in the study population (Delahay, Langton, Smith, Clifton‐Hadley, & Cheeseman, 2000), further investigation of the potential impact of seasonal forcing on infection may be highly informative. This is especially true as climate change may have the potential to alter these patterns, as weather within seasons can alter badger behavior (e.g., Noonan et al., 2014, 2015) and therefore may have the potential to influence disease dynamics. Such work might also consider the role of synchronized breeding in badgers (cubs being born in late winter) in driving seasonal changes in social contact patterns and disease dynamics. Changes in social network structure, for example as documented by Weber et al. (2013), may also contribute further to any role for social behavior in seasonal forcing of infection.

## 5 CONCLUSIONS

Understanding the impact of social behavior on pathogen dynamics in wildlife populations often requires a consideration of daily and seasonal variation in potentially infectious contact events (Altizer et al., 2006; Hamede et al., 2009; Hirsch et al., 2016), as well as its indirect impact on disease transmission through social buffering against infection risk (Ezenwa, Ghai, McKay, & Williams, 2016). In the present study, we have demonstrated important variation in daily and seasonal patterns of social contacts in badgers which may in turn drive seasonality in relationship between social behavior and disease risk. The results of this study suggest that evidence‐based models of pathogen ecology should consider seasonal variations in contact patterns even in situations where individuals appear to have relatively stable numbers of contacts. Seasonality in the nature of social interactions and subsequent forcing of infection could help explain complex spatio‐temporal patterns in disease occurrence observed in social species, as well as having the potential to result in changes to disease epidemiology in response to climate change.

## CONFLICT OF INTEREST

None declared.

## AUTHOR CONTRIBUTIONS

Data were collected by NW, RJD, and RAM. MJS, RJD, and RAM devised the study. Analysis was conducted by MJS and LCS with assistance from DPC and DJH. All authors contributed to the writing of the manuscript.
