# Supplementary material for: Seasonal variation in daily patterns of social contacts in the European badger Meles meles
Source: Ecol Evol. 2017 Sep 25;7(21):9006–15. doi: 10.1002/ece3.3402 (PMC5677474; doi:10.1002/ece3.3402)
Supplement: Supplementary file 1 [file ECE3-7-9006-s001.docx]

**Supplementary Results**

**Table S1.** A summary of model results for models of the probability of within-group interactions occurring and frequency of interactions when did occur using month rather than season as a fixed effect.

| **Model** | **Model terms** | **Type of effect** | **Effect** | **Direction** | **Significance** |
| --- | --- | --- | --- | --- | --- |
| Contact probability | Month | *Fixed* | 🗶 | NA | Χ^2^_(11)_=5.72  p=0.89 |
|  | Social Group | *Fixed* | 🗶 | NA | Χ^2^_(5)_=2.89  p=0.72 |
|  | **Sampling Effort (days both individuals collared)** | ***Fixed*** | **✓** | **+** | **Χ^2^_(1)_=6.46**  **p=0.01** |
|  | Length of time collared (days since collar deployment) | *Fixed* | 🗶  🗶 | NA  NA | Χ^2^_(1)_=3.18  p=0.07  Χ^2^_(1)_=2.28  p=0.13 |
|  | Individual ID 1 | *Random* | - | NA | - |
|  | Individual ID 2 | *Random* | - | NA | - |
| Contact frequency | Month | *Fixed* | **✓** | NA | Χ^2^_(11)_=85.82  p<0.001 |
|  | Social Group | *Fixed* | 🗶 | NA | Χ^2^_(5)_=4.45  p=0.49 |
|  | Sampling Effort (days both individuals collared) | *Fixed* | **✓** | **-** | **Χ^2^_(1)_=4.83**  **p=0.03** |
|  | Length of time collared (days since collar deployment) | *Fixed* | **✓**  🗶 | **-**  NA | **Χ^2^_(1)_=11.14**  **p<0.001**  Χ^2^_(1)_=2.68  P=0.10 |
|  | Individual ID 1 | *Random* | - | NA | - |
|  | Individual ID 2 | *Random* | - | NA | - |

**Table S2.** Model predictions for the effect of Month on the probability of within-group interactions and their mean daily frequency if they do occur. Predictions are back-transformed model estimates with standard errors, and are for dyads in group one simultaneously collared for 90 days of a season and for a time since collared of zero days.

| **Month** | **Mean probability of a contact event** | **Mean daily frequency of contacts** |
| --- | --- | --- |
| January | 0.80 (0.47-0.94) | 1.34 (0.61-2.92) |
| February | 0.81 (0.48-0.95) | 0.62 (0.28-1.38) |
| March | 0.83 (0.52-0.96) | 0.47 (0.21-1.06) |
| April | 0.82 (0.49-0.96) | 0.49 (0.21-1.14) |
| May | 0.86(0.55-0.97) | 0.55 (0.23-1.33) |
| June | 0.85 (0.59-0.96) | 3.91 (1.88-8.09) |
| July | 0.83 (0.56-0.95) | 2.53 (1.22-5.25) |
| August | 0.85 (0.59-0.96) | 1.62 (0.78-3.36) |
| September | 0.82 (0.53-0.95) | 1.01 (0.48-2.11) |
| October | 0.82 (0.53-0.95) | 0.88 (0.42-1.86) |
| November | 0.81 (0.51-0.95) | 1.17 (0.55-2.48) |
| December | 0.77 (0.44-0.93) | 1.42 (0.66-3.08) |


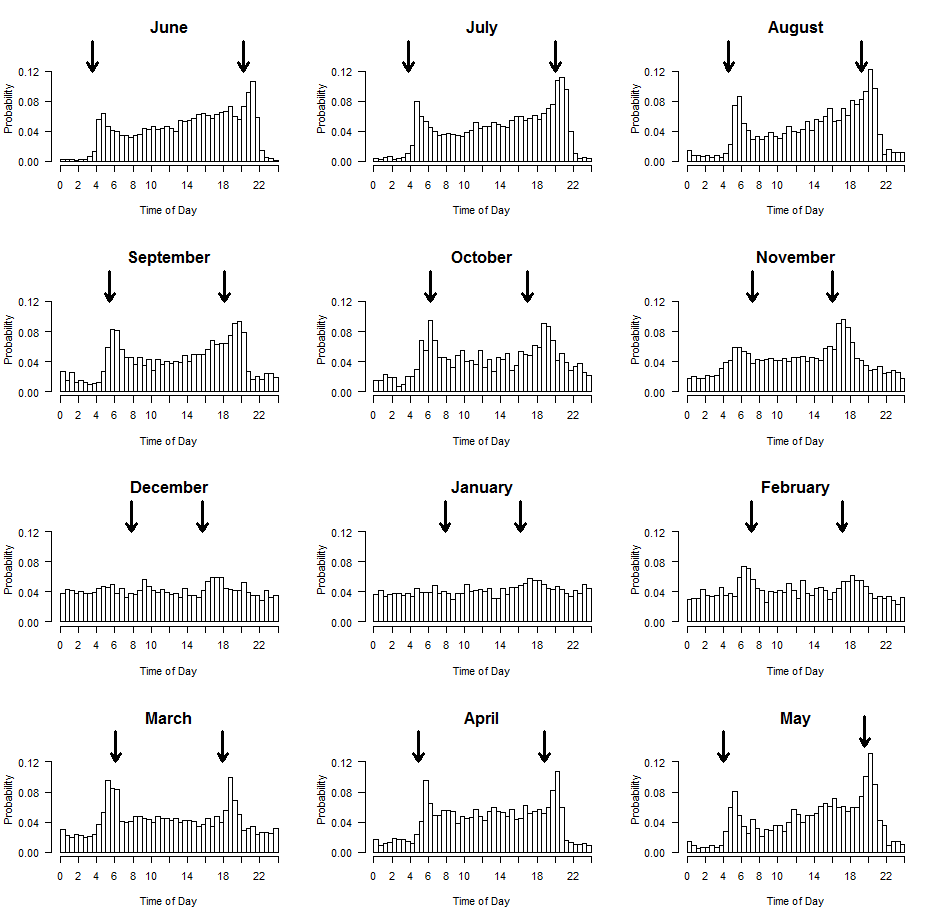


**Figure S1.** Variation among months in the daily pattern of within-group contacts of badgers. The arrows show sunrise and sunset times on the middle day of each season. Each day is split into 30 minute intervals. The year starts with summer to correspond with deployment of the first collars.


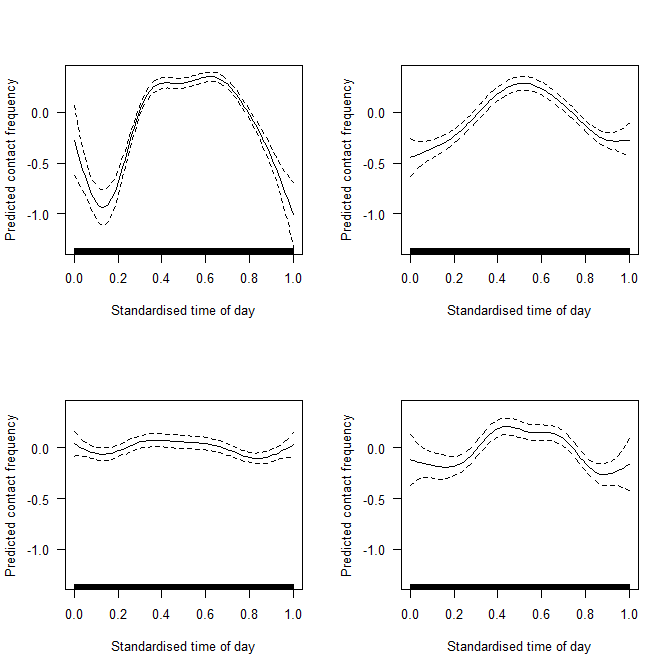


**Figure S2.** Plots extracted from a general additive model of the relationship between contact duration and time of day, with time of day standardised so that 25% of the day happens before sunrise, 50% between sunrise and sunset and 75% after sunset. Plots are for a) summer, b) autumn, c) winter and d) spring. The model includes fixed effects for each individual involved in a contact event and social group, as well as the interaction between season and time of day. The level of smoothing was determined by the model.


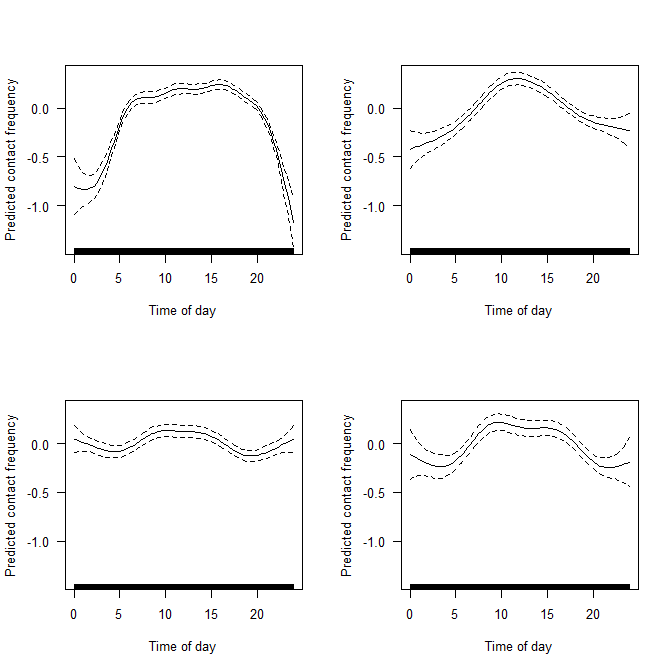


**Figure S3.** Plots extracted from a general additive model of the relationship between contact duration and time of day. Plots are for a) summer, b) autumn, c) winter and d) spring. The model includes fixed effects for each individual involved in a contact event and social group, as well as the interaction between season and time of day. The level of smoothing was determined by the model.
